# Baseline, Early Changes, and Residual Albuminuria: *Post Hoc* Analysis of a Randomized Clinical Trial of Dapagliflozin in Chronic Kidney Disease

**DOI:** 10.2215/CJN.0000000000000550

**Published:** 2024-12-09

**Authors:** Dominique van Mil, Priya Vart, Glenn M. Chertow, Ron T. Gansevoort, Peter Rossing, Robert D. Toto, Ricardo Correa-Rotter, Anna Maria Langkilde, C. David Sjöström, David C. Wheeler, Hiddo J.L. Heerspink

**Affiliations:** 1 Department of Clinical Pharmacy and Pharmacology, University Medical Center Groningen, University of Groningen, Groningen, The Netherlands; 2 Department of Internal Medicine, University Medical Center Groningen, University of Groningen, Groningen, The Netherlands; 3 Departments of Medicine, Epidemiology and Population Health, and Health Policy, Stanford University School of Medicine, Stanford, California; 4 Steno Diabetes Center Copenhagen, Gentofte, Denmark; 5 Department of Clinical Medicine, University of Copenhagen, Copenhagen, Denmark; 6 Department of Internal Medicine, UT Southwestern Medical Center, Dallas, Texas; 7 The National Medical Science and Nutrition Institute Salvador Zubiran, Mexico City, Mexico; 8 Late-Stage Development, Cardiovascular, Renal, and Metabolism, BioPharmaceuticals R&D, AstraZeneca, Gothenburg, Sweden; 9 Department of Renal Medicine, University College London, London, United Kingdom; 10 The George Institute for Global Health, Sydney, New South Wales, Australia

**Keywords:** albuminuria, cardiovascular events, CKD, diabetes, ESKD, GFR, outcomes, randomized controlled trials, risk factors, SGLT2

## Abstract

**Key Points:**

- Dapagliflozin reduced albuminuria in participants with CKD, with reductions being proportional to reductions in the risk of disease progression.
- Residual albuminuria (at month 4) was linked to higher risks of primary and kidney end points, with no heterogeneity by diabetes status or allocated treatment.
- Participants with residual albuminuria at month 4 had high rates of kidney end points, implying the need for added therapy for long-term kidney and cardiovascular benefits.

**Background:**

Albuminuria is a strong indicator of kidney and cardiovascular risk in patients with CKD. We assessed risk associations between albuminuria at baseline and 4 months after randomization in a placebo-controlled trial of dapagliflozin and kidney end points in patients with CKD and albuminuria, with and without type 2 diabetes.

**Methods:**

In this *post hoc* analysis of the dapagliflozin and prevention of adverse outcomes in CKD (DAPA-CKD) trial, 4304 adult patients with CKD were randomized to dapagliflozin 10 mg or placebo as an adjunct to maximally tolerated renin-angiotensin system inhibitors. The primary end point was a composite of sustained ≥50% decline in eGFR, kidney failure, or death from kidney or cardiovascular cause. The kidney composite end point was similar, but excluded cardiovascular death. We assessed associations among baseline albuminuria, early change in albuminuria (baseline to month 4), and residual albuminuria (month 4) with the primary composite and kidney composite end points using Cox proportional hazards regression analyses.

**Results:**

Compared with placebo, dapagliflozin reduced urinary albumin–creatinine ratio (baseline to month 4) by 36.4% (95% confidence interval, 30.2% to 42.5%) and 20.5% (95% confidence interval, 11.6% to 29.5%) in participants with and without type 2 diabetes, respectively (*P*-interaction: 0.02). A reduction in urinary albumin–creatinine ratio from baseline to month 4 was associated with a lower risk of the primary and kidney composite end points with a similar risk gradient for participants with and without type 2 diabetes (*P*-interaction: 0.10 and 0.19, respectively). Residual albuminuria was associated with a similar risk of the primary and kidney composite end points in each treatment arm (*P*-interaction: 0.19 and 0.18, respectively).

**Conclusions:**

Dapagliflozin reduced albuminuria, and the magnitude of albuminuria reduction showed similar proportional reductions in risks of the primary and kidney composite end points in participants with and without type 2 diabetes. Participants with residual albuminuria at month 4—whether randomized to dapagliflozin or placebo—experienced relatively high rates of CKD progression kidney end points, suggesting that therapies added to renin-angiotensin system inhibitors and dapagliflozin may be required to sustain kidney and cardiovascular health.

**Clinical trial registry name and registration number:**

A Study to Evaluate the Effect of Dapagliflozin on Renal Outcomes and Cardiovascular Mortality in Patients with CKD (DAPA-CKD), NCT03036150.

## Visual Abstract

**Figure:**
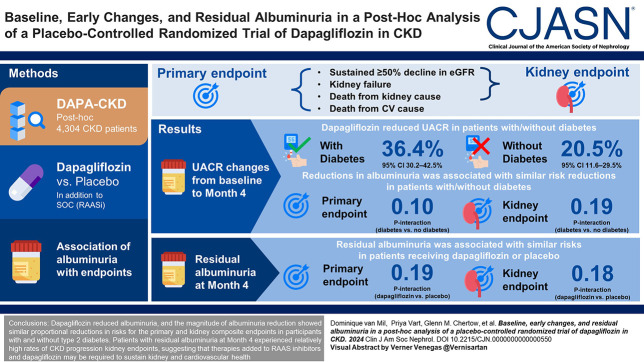

## Introduction

Albuminuria is a strong marker for kidney and cardiovascular risk in patients with type 2 diabetes or CKD.^1–5^ Sodium–glucose co-transporter 2 (SGLT2) inhibitors attenuate progressive loss of kidney function and reduce the risk of kidney failure in patients with CKD and type 2 diabetes.^6^ SGLT2 inhibitors also reduce albuminuria in patients with type 2 diabetes and cardiovascular disease or CKD.^6–8^ The reduction in albuminuria is seen early after SGLT2 inhibitor initiation and is sustained during prolonged treatment. A *post hoc* analysis from the canagliflozin and renal events in diabetes with established nephropathy clinical evaluation trial showed that in patients with type 2 diabetes and CKD, the early reduction in albuminuria explained 48% of the effect of canagliflozin in reducing the risk of kidney failure.^9^ These data suggest that in patients with type 2 diabetes, the early change in albuminuria during SGLT2 inhibition explained part of the long-term clinical benefit and may be used to monitor long-term prognosis.

The SGLT2 inhibitors dapagliflozin and empagliflozin reduced albuminuria and the decline in eGFR in patients with CKD with and without type 2 diabetes on the basis of evidence from dapagliflozin and prevention of adverse outcomes in CKD (DAPA-CKD), a study to evaluate the effect of dapagliflozin on renal outcomes and cardiovascular mortality in patients with CKD, and the Study of Heart and Kidney Protection with Empagliflozin trials.^10–12^ Detailed analyses from the DAPA-CKD trial demonstrated that the benefits of dapagliflozin in reducing progressive kidney disease and heart failure or cardiovascular death were evident across the spectrum of baseline albuminuria and KDIGO risk categories.^13,14^ Whether early SGLT2 inhibitor–induced changes in albuminuria are associated with kidney or cardiovascular events in patients with CKD without type 2 diabetes is unknown.

We conducted a *post hoc* analysis of the DAPA-CKD trial to examine risk associations between albuminuria and kidney end points before and during treatment with dapagliflozin or placebo in patients with CKD with and without type 2 diabetes.

## Methods

### Study Design and Participants

The DAPA-CKD trial was a double-blind, randomized, placebo-controlled, multicenter clinical trial that recruited participants in 21 countries at 386 study sites between February 2017 and June 2020. The study design and main results have been published separately.^10,15^ The study protocol was approved by a central or local ethics committee at each trial site, and all participants provided written informed consent before any study procedure was performed. The DAPA-CKD trial is registered with ClinicalTrials.gov (NCT03036150).

In short, patients aged 18 years or older with a diagnosis of CKD (defined as eGFR 25–75 ml/min per 1.73 m^2^ and urinary albumin–creatinine ratio [UACR] 200–5000 mg/g) with or without type 2 diabetes were eligible for participation. Patients were excluded in case of documented diagnosis of type 1 diabetes, polycystic kidney disease, lupus nephritis, anti-neutrophil cytoplasmic antibody–associated vasculitis, or receiving immunotherapy for primary or secondary kidney disease within 6 months before enrollment. Prescription of a stable maximum labeled or maximum tolerated dose of an angiotensin-converting enzyme inhibitor or angiotensin-receptor blocker for 4 weeks or longer was required before random assignment to treatment unless there was documented intolerance to these drugs.

### Procedures

Eligible participants were randomly assigned (1:1) to receive dapagliflozin 10 mg once daily or matching placebo, in addition to standard care. Randomization was stratified by diagnosis of type 2 diabetes and UACR as ≤1000 mg/g or >1000 mg/g. Study treatment was discontinued in case of diabetic ketoacidosis, pregnancy, receipt of disallowed therapy, or study completion. Follow-up study visits were performed at 2 weeks; 2, 4, and 8 months; and thereafter at 4-month intervals after randomization. At each study visit, vital signs and information on potential study end points, adverse events, concomitant therapies, and study drug adherence were recorded. Urine and blood samples were obtained for laboratory assessment. Participants and study personnel were masked to treatment allocation.

### Albuminuria Measurements

Urinary albumin and creatinine were measured in single urine specimens from the first morning void. Albuminuria was expressed as urinary albumin divided by urinary creatinine, thus the UACR. We defined UACR at baseline as the mean of the UACR values from samples collected at the screening and randomization visits measured in a central laboratory. Early change in albuminuria was defined as the proportion change in UACR between baseline (before start of the assigned study treatment) and 4 months (16 weeks) of using the assigned study treatment. UACR at each study visit was measured in a single first morning void sample. The 16-week window was chosen because prior studies have indicated that the UACR-lowering effect of SGLT2 inhibitors is fully present by that point in time.^12^

### Outcomes

The primary end point of the DAPA-CKD trial was a composite of the occurrence of a decline ≥50% in eGFR (confirmed by a second serum creatinine measurement after at least 28 days), ESKD (defined as initiation of maintenance dialysis, kidney transplantation, or eGFR <15 ml/min per 1.73 m^2^), or death from a kidney or cardiovascular cause. Secondary end points included a kidney composite end point (comprising the primary end point, but excluding cardiovascular death), a cardiovascular composite end point comprising hospitalized heart failure or cardiovascular death, and all-cause mortality. All clinical end points were assessed by an independent, blinded, event adjudication committee using the predefined and rigorous end point definitions. For the current analyses, we focused on the primary and kidney composite end points.

### Statistical Analyses

Baseline characteristics were summarized by categories of early change in UACR at month 4. We report continuous variables as mean (SD) or median (25%–75% range) in case of skewed distributions. Categorical variables were presented as *n* (%). Early change in UACR from baseline to month 4 was expressed as percentage in four groups. We applied thresholds of 30% as the definition of change in UACR. Large-scale meta-analyses of both observational and randomized studies have demonstrated that 30% changes in UACR associate firmly with kidney outcomes and treatment effects on kidney outcomes, respectively.^16,17^

First, we investigated the associations of baseline UACR with the primary and kidney composite end points. For these analyses, we used Cox proportional hazards regression, with baseline UACR fitted continuously, comparing participants with and without type 2 diabetes. The model was adjusted for the baseline covariates of randomized treatment, age, sex, designated race, eGFR, current smoking status, Quételet (body mass) index (BMI), systolic BP, hemoglobin A1c (HbA1c), hemoglobin, and cardiovascular disease history. We stratified the analysis by presence or absence of type 2 diabetes using a UACR reference point of 500 mg/g, to show interaction with type 2 diabetes. To plot the association of UACR with clinical outcomes by diabetes status when using UACR of 500 mg/g in the subgroup of participants without type 2 diabetes as a reference, UACR was first fitted categorically and, subsequently, hazard ratios (HR) were plotted using a locally weighted scatterplot smoothing procedure (*i*.*e*., Lowess smoothing).

Second, when assessing the associations between the early change in UACR and the primary and kidney composite end points, we followed participants starting at month 4 until the first event of the primary or kidney composite end point, death, or end of follow-up, fitting the model with change in UACR as a continuous variable and excluding participants if they had reached the primary or kidney composite end point before month 4. We adjusted for randomized treatment, age, sex, designated race, eGFR, smoking status, BMI, systolic BP, HbA1c, hemoglobin, cardiovascular disease history, and log-transformed UACR from baseline. We plotted hazard ratios for participants stratified by presence or absence of type 2 diabetes, using a UACR change of 0% as a reference to show the interaction with type 2 diabetes.

In addition, to further examine the association of change from baseline in UACR at month 4 and the remaining UACR level at month 4 (hereafter referred to as residual UACR), with the primary and kidney composite end points by treatment groups, we applied another regression model, adjusting for baseline covariates age, sex, designated race, eGFR, smoking status, BMI, systolic BP, HbA1c, hemoglobin, diabetes, and cardiovascular disease history, all at baseline. To plot the association of UACR with clinical end points by treatment groups when using UACR in the placebo group as reference, UACR was first fitted categorically and, subsequently, HRs were plotted using Lowess smoothing. A residual UACR of 500 mg/g at month 4 in the placebo group was used as a reference. We also performed analyses stratified by presence or absence of type 2 diabetes.

To further examine the associations of UACR with the primary and kidney composite end points, we conducted a companion analysis wherein we considered UACR as a time-dependent covariate, adjusting for the same baseline covariates listed above. Finally, to investigate whether the association of the residual albuminuria with the primary and kidney composite end points were dependent on baseline UACR, we fitted our model adjusting for baseline UACR and examined the interaction between baseline and month 4 residual albuminuria.

Two-tailed *P* values of <0.05 were considered to be statistically significant. All analyses were performed using Stata 17 (StataCorp).

## Results

In total, 4304 participants were enrolled and randomly assigned to dapagliflozin 10 mg (*n*=2152) or placebo (*n*=2152) once daily.^10^ The median follow-up was 2.4 (25%–75% range: 2.0–2.7) years. Of the 4304 participants in the DAPA-CKD trial, 364 lacked information on UACR at month 4, experienced a primary or kidney composite end point between baseline and month 4, or were censored. The final study population for early change in UACR included 3940 participants (91.5%).

### Association between Baseline UACR and Primary and Kidney Composite End Points

**Figure 1:**
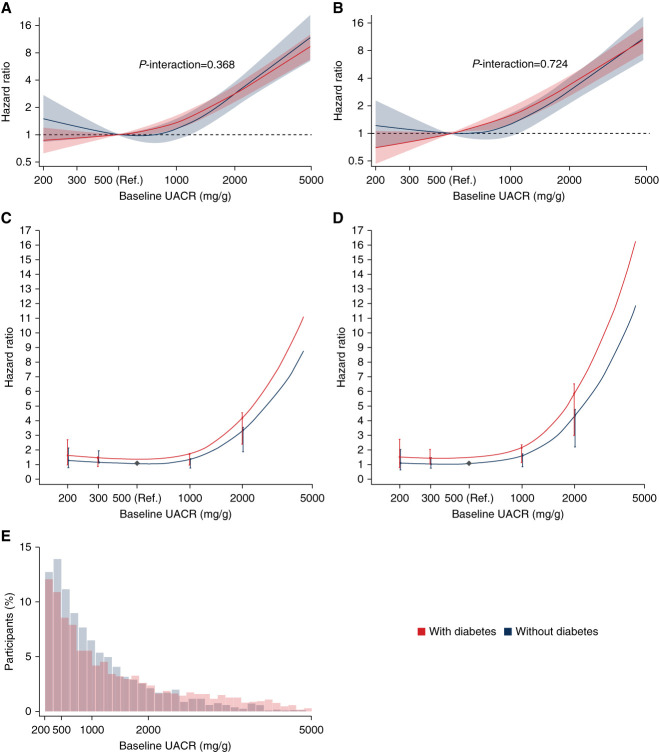
**Association of baseline albuminuria with primary and kidney end points in participants with and without type 2 diabetes**. (A and B) Association of baseline albuminuria with primary (A) and kidney (B) end points, using a UACR reference point of 500 mg/g in the subgroups of participants with and without type 2 diabetes separately. (C and D) Association of baseline albuminuria with primary and kidney end points, respectively, using a common reference point (UACR of 500 mg/g in the subgroup of participants without type 2 diabetes; vertical lines represent 95% CI). (E) Distribution of the baseline UACR by number of participants separately with and without type 2 diabetes. CI, confidence interval; Ref, reference; UACR, urinary albumin–creatinine ratio.

The risks of the primary and kidney composite end points were higher among participants with higher baseline UACR (Figure 1, A and B). The associations between baseline UACR and the primary and kidney composite end points were similar for participants with and without type 2 diabetes (*P*-interaction for the primary and kidney end points: 0.37 and 0.72, respectively) indicating that each increment in UACR carried the same relative risk in participants with or without type 2 diabetes. When using one reference point in the group of participants without type 2 diabetes to determine the risk for participants with type 2 diabetes, the HR for the primary and kidney composite end points at a given UACR level was higher for participants with type 2 diabetes compared with those without type 2 diabetes, illustrating the role of type 2 diabetes itself on the risk profile (Figure 1, C and D). Most of the participants had a baseline UACR between 200 and 1000 mg/g (Figure 1E).

### Association between Early Change in UACR and Primary and Kidney Composite End Points

A reduction in UACR of >30% was observed in 1913 participants (48.6%), a lesser reduction of 0 to ≤30% in 763 (19.4%), a modest increase of 0% to <30% in 617 (15.7%), and a ≥30% increase in 647 (16.4%). Table 1 summarizes the characteristics of participants stratified according to early change in UACR from baseline to month 4. Participants with a >30% reduction in UACR were older; more often female; had higher baseline systolic BP and eGFR; and were more likely to be diagnosed with type 2 diabetes, cardiovascular disease, and heart failure compared with those with a modest reduction or increase in UACR (Table 1). Notably, participants treated with dapagliflozin were more likely to experience a >30% reduction in UACR. Participants with a ≥30% increase in UACR had the lowest baseline UACR and hemoglobin. When stratified according to early change in UACR from baseline to month 4 and treatment status, participants with a >30% reduction in UACR and randomized to dapagliflozin, were more likely to have a history of type 2 diabetes and diagnosis of diabetic kidney disease and higher baseline UACR, compared with those with a >30% reduction in UACR and randomized to placebo (Supplemental Table 1). This was also the case when early change in UACR was ranked into quartiles (Supplemental Table 2).

**Table 1:** Baseline characteristics of participants by early change in albuminuria at month 4

Characteristics	Early Change in Albuminuria at Month 4				*P* Value
	>30% Decrease	0% to ≤30% Decrease	0% to <30% Increase	≥30% Increase	
*n* (%)	1913 (48.6)	763 (19.4)	617 (15.7)	647 (16.4)	
UACR change in % at month 4, median (IQR)	−77.0 (−118.7 to−50.5)	−15.4 (−22.9 to−7.4)	13.0 (5.9 to 21.1)	58.6 (41.9 to 83.6)	<0.001
Baseline UACR, mg/g, median (IQR)	951 (466–1801)	1054 (521–2127)	1049 (481–2224)	779 (415–1471)	<0.001
**UACR baseline category, mg/g, *n* (%)**					<0.001
<300	206 (10.8)	62 (8.1)	55 (8.9)	92 (14.2)	
≥300 to <1000	787 (41.1)	302 (39.6)	242 (39.2)	305 (47.1)	
≥1000 to <3000	735 (38.4)	302 (39.6)	219 (35.5)	204 (31.5)	
≥3000	185 (9.7)	97 (12.7)	101 (16.4)	46 (7.1)	
Age, yr	63.2 (11.3)	61.9 (12.2)	60.4 (13.0)	60.6 (12.3)	<0.001
Men, *n* (%)	1228 (64.2)	553 (72.5)	425 (68.9)	433 (66.9)	<0.001
**Race, *n* (%)**					<0.001
Asian	519 (27.1)	255 (33.4)	197 (31.9)	239 (36.9)	
Black	103 (5.4)	27 (3.5)	24 (3.9)	26 (4.0)	
Other	178 (9.3)	68 (8.9)	47 (7.6)	44 (6.8)	
White	1113 (58.2)	413 (54.1)	349 (56.6)	338 (52.2)	
Current smoker, *n* (%)	232 (12.1)	113 (14.8)	91 (14.8)	81 (12.5)	0.16
**CKD diagnosis, *n* (%)**					<0.001
Diabetic nephropathy	1168 (61.1)	440 (57.7)	346 (56.1)	380 (58.7)	
Hypertensive CKD	314 (16.4)	129 (16.9)	107 (17.3)	96 (14.8)	
GN	235 (12.3)	131 (17.2)	115 (18.6)	118 (18.2)	
Other or unknown	196 (10.3)	63 (8.3)	49 (7.9)	53 (8.2)	
History of diabetes, *n* (%)	1339 (70.0)	518 (67.9)	394 (63.9)	446 (68.9)	0.04
Duration of diabetes, yr, median (IQR)*	13.8 (7.4–20.8)	14.1 (7.6–20.5)	15.1 (8.0–20.8)	12.7 (6.7–20.2)	0.23
History of cardiovascular disease, *n* (%)	775 (40.5)	273 (35.8)	209 (33.9)	247 (38.2)	0.01
History of heart failure, *n* (%)	243 (12.7)	72 (9.4)	50 (8.1)	81 (12.5)	0.003
Weight, kg	82.4 (20.5)	83.0 (20.3)	82.7 (21.5)	81.6 (21.1)	0.66
BMI, kg/m^2^	29.9 (6.2)	29.5 (6.0)	29.8 (6.7)	29.5 (6.0)	0.37
Systolic BP, mm Hg	139.2 (17.7)	137.3 (16.9)	135.6 (17.6)	133.4 (15.9)	<0.001
Diastolic BP, mm Hg	77.7 (10.5)	77.7 (10.4)	77.6 (10.4)	76.5 (10.0)	0.08
HbA1c, %	7.1 (1.6)	7.1 (1.7)	7.0 (1.8)	7.2 (1.9)	0.08
eGFR, ml/min per 1.73 m^2^	44.0 (12.3)	42.6 (12.7)	42.7 (12.4)	43.0 (12.6)	0.02
Hemoglobin, g/L	128.7 (17.6)	129.2 (18.2)	127.6 (18.5)	126.3 (18.1)	0.009
Baseline ACE inhibitor/ARB use, *n* (%)	1888 (98.7)	750 (98.3)	601 (97.4)	630 (97.4)	0.06
Baseline diuretic use, *n* (%)	894 (46.7)	312 (40.9)	272 (44.1)	295 (45.6)	0.05
**Randomized treatment, *n* (%)**					<0.001
Dapagliflozin	1187 (62.1)	332 (43.5)	236 (38.3)	223 (34.5)	
Placebo	726 (38.0)	431 (56.5)	381 (61.8)	424 (65.5)	

Data are presented mean (SD), unless otherwise indicated. ACE, angiotensin-converting enzyme; ARB, angiotensin-receptor blocker; BMI, body mass index; HbA1c, hemoglobin A1c; IQR, interquartile range; n, number; UACR, urinary albumin–creatinine ratio.

* Among those participants with a history of diabetes.

**Figure 2:**
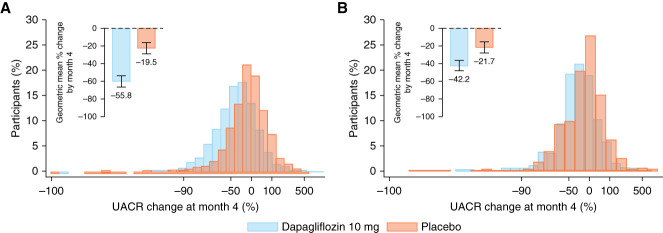
**Lowering of albuminuria by dapagliflozin versus placebo at month 4 in participants with and without type 2 diabetes.** (A) Distribution of the percentage of UACR change at month 4 in participants with type 2 diabetes, separately for the dapagliflozin and placebo groups. (B) Distribution of the percentage of UACR change at month 4 in participants without type 2 diabetes, separately for the dapagliflozin and placebo groups. The geometric mean percentage change in UACR is included in both panels (data are presented as mean [SD]). SD, standard deviation.

**Figure 3:**
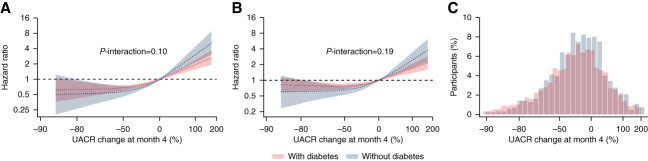
**Association of UACR change at 4 months of using the assigned study treatment with primary and kidney end points in participants with and without type 2 diabetes.** (A and B) Association of UACR change at month 4 with primary (A) and kidney (B) end points, using a reference point of UACR change of 0% in the subgroups of participants with and without type 2 diabetes separately. (C) Distribution of the percentage change in UACR at month 4 by number of participants with and without type 2 diabetes separately.

Compared with placebo, dapagliflozin reduced UACR at month 4 in participants with and without type 2 diabetes (Figure 2, A and B), but the effect was more pronounced in participants with type 2 diabetes (geometric mean percentage reduction: 36.4%, 95% confidence interval [CI], 30.2% to 42.5%) compared with those without (geometric mean percentage reduction: 20.5%, 95% CI, 11.6% to 29.5%; *P*-interaction: 0.02). Notably, there was considerable variation in UACR change among individual participants in the type 2 diabetes and no–type 2 diabetes subgroups (Figures 2, A and B and 3C).

The change in UACR from baseline to month 4 was associated with the primary and kidney composite end points (Figure 3, A and B) such that a larger reduction in UACR at month 4 was associated with a larger risk reduction for the primary and kidney composite end points. There was no evidence that the associations between change in UACR from baseline to month 4 and the primary and kidney composite end points differed among participants with and without type 2 diabetes (*P*-interaction: 0.10 and 0.19, respectively). Analyzing early change in UACR as quartiles showed a similar association as our main analyses (Supplemental Table 3). In addition, when analyzing UACR as a time-varying covariate, each doubling in UACR from baseline showed strong associations with the primary and kidney composite end point (HR, 1.62; 95% CI, 1.50 to 1.75 and HR, 1.92; 95% CI, 1.65 to 2.24 for the primary composite end point in participants with and without type 2 diabetes, respectively; HR, 2.02; 95% CI, 1.83 to 2.22 and HR, 2.19; 95% CI, 1.83 to 2.58 for the kidney composite end point in participants with and without type 2 diabetes, respectively).

### Association between Residual UACR and Primary and Kidney Composite End Points

In participants with and without type 2 diabetes, residual UACR at month 4 was associated with the primary and kidney composite end points (HR, 2.03; 95% CI, 1.73 to 2.38 and HR, 2.54; 95% CI, 1.93 to 3.35 for the primary composite end point, in participants with and without type 2 diabetes, respectively; HR, 2.33; 95% CI, 1.94 to 2.79 and HR, 2.93; 95% CI, 2.18 to 3.94 for the kidney composite end point in participants with and without type 2 diabetes, respectively). Moreover, the association between albuminuria and the primary and kidney end points depended on both the baseline and residual albuminuria (Supplemental Figure 1).

**Figure 4:**
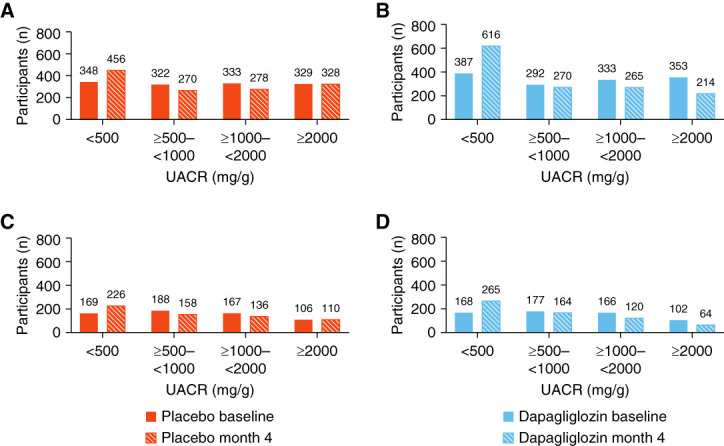
**Total number of participants per UACR category at baseline and with residual UACR at month 4.** (A and C) Total number of participants per UACR category at baseline and month 4 for participants with and without type 2 diabetes, respectively, randomized to placebo treatment. (B and D) Total number of participants per UACR category at baseline and at month 4 for participants with and without type 2 diabetes, respectively, randomized to dapagliflozin treatment.

Comparing the total number of participants in each UACR category at baseline and month 4, more participants in the dapagliflozin group (Figure 4, B and D) were categorized into the lower UACR categories at month 4, compared with the placebo group (Figure 4, A and C). This effect was more prominent among participants with type 2 diabetes (Figure 4, A and B), compared with those without type 2 diabetes (Figure 4, C and D). Although dapagliflozin significantly reduced UACR, a sizable proportion of dapagliflozin-treated participants continued to exhibit UACR levels ≥1000 mg/g at month 4: 35.1% of those with type 2 diabetes and 30.0% of those without type 2 diabetes (Figure 4, B and D).

### Risks Associated with Albuminuria by Randomized Treatment Assignment

**Figure 5:**
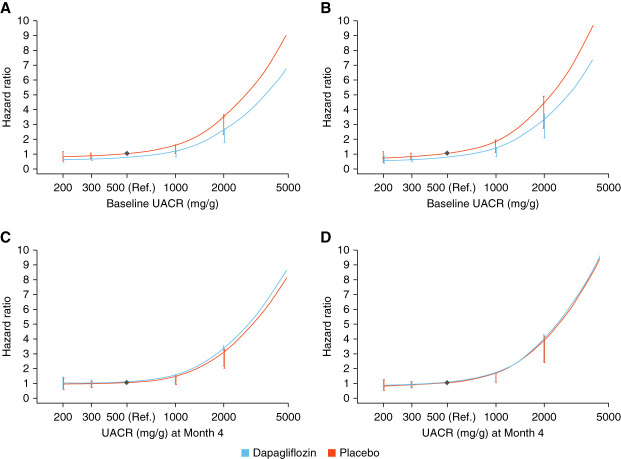
**Association of baseline albuminuria with primary and kidney end points and association of residual albuminuria at month 4 with primary and kidney end points in patients randomized to placebo or dapagliflozin.** (A and B) Association of baseline albuminuria with primary and kidney end points, respectively, using a reference point of baseline UACR of 500 mg/g in the placebo treatment group. (C and D) Association of the residual albuminuria at month 4 with primary and kidney end points, respectively, using a reference point of residual UACR of 500 mg/g at month 4 in the placebo treatment group. Vertical lines represent 95% CI.

When analyzing the dapagliflozin and placebo groups stratified by diabetes status, the relative hazards linking baseline albuminuria with the primary and kidney composite end points were attenuated in dapagliflozin-treated participants (Figure 5, A and B). By contrast, the relative hazards of residual UACR at month 4 for the primary and kidney composite end points were similar in dapagliflozin-treated and placebo-treated participants, suggesting that residual UACR in the dapagliflozin-treated participants carried similar risk as in the placebo-treated participants (*P*-interaction: 0.19 and 0.18, respectively, for the primary and kidney end point; Figure 5, C and D). These patterns of risk were similar among participants with and without type 2 diabetes (Supplemental Figure 2). As might be expected, participants with a higher level of residual albuminuria at month 4 had a higher baseline UACR compared with those with lower ratios or residual albuminuria (Supplemental Table 4).

## Discussion

In this *post hoc* analysis of the DAPA-CKD trial, we found that higher baseline albuminuria was associated with a higher risk of progressive kidney disease or cardiovascular death. The magnitude of albuminuria reduction was proportional to the reduction in risks of the primary and kidney composite end points, with no evidence of heterogeneity by type 2 diabetes status. Dapagliflozin reduced albuminuria to a larger degree among participants with type 2 diabetes compared with participants without type 2 diabetes. Residual albuminuria (on the basis of values at month 4) was associated with higher risks of the primary and kidney composite end points, with no evidence of heterogeneity by type 2 diabetes status or randomized treatment allocation. The latter observation suggests that despite sizable average reductions in albuminuria and dramatic reductions in the risk of progressive kidney disease or cardiovascular death, there are subpopulations of patients (with and without type 2 diabetes) who continue to exhibit progressive kidney disease despite treatment with dapagliflozin.

The finding that participants with larger relative reductions in albuminuria experienced more favorable outcomes compared with participants in whom the albuminuria levels did not change or increase confirms and extends findings from *post hoc* analyses of several other clinical trials of SGLT2 inhibitors in patients with type 2 diabetes.^9,18–20^ Although dapagliflozin reduced the risk of clinical end points in participants with and without type 2 diabetes, the observation that albuminuria was reduced to a larger degree in those with type 2 diabetes suggests that the effect of dapagliflozin is mediated through different pathways and that its albuminuria-reducing effects may be more potent among patients with type 2 diabetes than among patients with other kidney diseases. However, the DAPA-CKD trial was not designed to investigate the mechanisms of action of dapagliflozin, and additional research is needed to explore the multiple potential mechanisms for kidney protection in patients with versus without type 2 diabetes.

Our findings related to residual albuminuria may have the greatest relevance to ongoing clinical practice. Participants with or without type 2 diabetes with residual high-grade albuminuria at month 4—whether randomized to dapagliflozin or placebo—experienced relatively higher rates of kidney end points. The finding that residual albuminuria remained associated with clinical end points when adjusting for the baseline UACR implies that higher residual albuminuria is associated with poorer prognosis irrespective of the starting UACR and highlights the relevance of monitoring UACR. Our results further suggest that there is a subpopulation of patients (with and without type 2 diabetes) in whom the effect of dapagliflozin is insufficient to lower albuminuria and prevent progressive kidney disease. In patients with high-grade residual albuminuria, additional albuminuria-lowering therapies should be considered that might augment the benefit afforded by renin-angiotensin system (RAAS) inhibitors and dapagliflozin. In patients with type 2 diabetes and CKD, the nonsteroidal mineralocorticoid receptor antagonist, finerenone, has been shown to yield kidney and cardiovascular benefits.^21,22^ Favorable effects of endothelin receptor antagonists have been demonstrated in diabetic kidney disease and IgA nephropathy ^23^; the role for these agents in patients jointly treated with RAAS inhibitors and SGLT2 inhibitors is currently being investigated in the Combination Effect of Finerenone and Empagliflozin in Participants with CKD and Type 2 Diabetes Using a UACR Endpoint and Zibotentan and Dapagliflozin for the Treatment of CKD clinical trials.^24,25^ Several studies have suggested albuminuria-reducing effects of glucagon-like peptide-1 (GLP-1) receptor agonists and glucose-dependent insulinotropic polypeptide–GLP-1 combination receptor agonists.^26^ The Evaluate Renal Function with Semaglutide Once Weekly trial is the first dedicated kidney outcome trial with a GLP-1 receptor agonist that recently showed the benefit of semaglutide on kidney outcomes in patients with CKD and type 2 diabetes.^27^ Finally, several immunomodulatory agents in development for IgA nephropathy have demonstrated clear proteinuria-reducing effects.^28^ These therapies may have a role more broadly in diabetic and nondiabetic CKD.

There are several strengths of our analysis, primarily related to the rigor required of data collection in large-scale clinical trials. Nearly all participants had available data on albuminuria at month 4 so that associations between the change in albuminuria (from baseline to month 4) and residual albuminuria (at month 4) with end points could be explored. The trial population was diverse by age, sex, designated race and ethnicity, geography, and underlying etiology of CKD. Limitations include the possibility of misclassification of the level of albuminuria (at baseline and at month 4), which could bias the associations toward the null. Albuminuria was measured in single first morning void urine samples, which increases the likelihood of random fluctuations compared with guideline-recommended assessment in three consecutive first morning void samples. In addition, albuminuria is widely variable within individuals over time, as was apparent in the placebo group, which should be kept in mind when monitoring UACR over time. Moreover, while the trial included a broad range of participants across the world, they were all clinical trial participants and may be different in fundamental ways from patients seen in clinical practice. Because the DAPA-CKD trial included participants with a baseline UACR above 200 mg/g, no conclusions can be drawn for patients with lower levels of baseline albuminuria. In addition, because of the relatively short follow-up, relatively few kidney end points were observed among participants with baseline UACR between 200 and 1000 mg/g, limiting the precision of the effect estimates. Larger observational studies with longer follow-up demonstrated consistent linear associations with no apparent UACR threshold below which this association is attenuated.^5,29^ This was a *post hoc* analysis, and we cannot exclude chance findings. In addition, we do not make inferences that changes that occurred in albuminuria were due to the effect of treatment, because a parallel randomized placebo-controlled trial does not enable differentiation of true changes in albuminuria from changes due to regression to the mean or natural disease progression. Finally, the DAPA-CKD trial was terminated early because of efficacy; thus, follow-up time was truncated, and the overall number of events was less than anticipated.

In summary, among patients with and without type 2 diabetes entering a randomized placebo-controlled clinical trial with eGFR 25–75 ml/min per 1.73 m^2^ and UACR 200–5000 mg/g, higher levels of albuminuria at baseline were associated with higher risks of the primary and kidney composite end points. Larger reductions in UACR (from baseline to month 4) were associated with lower risks of the primary and kidney composite end points. Dapagliflozin reduced albuminuria, and the magnitude of albuminuria reduction was associated with similar proportional reductions in risk of the primary and kidney end points in participants with and without type 2 diabetes. In addition, participants with residual high-grade albuminuria at month 4—whether randomized to dapagliflozin or placebo—experienced similar and relatively high rates of kidney end points, suggesting that therapies added to RAAS inhibitors and dapagliflozin might be required to improve long-term kidney and cardiovascular prognosis.

## Supplementary Material

## Data Availability

Data underlying the findings described in this manuscript may be obtained in accordance with AstraZeneca's data sharing policy described at: https://astrazenecagrouptrials.pharmacm.com/ST/Submission/Disclosure. Data for studies directly listed on Vivli can be requested through Vivli at www.vivli.org. Data for studies not listed on Vivli could be requested through Vivli at https://vivli.org/members/enquiries-about-studies-not-listed-on-the-vivli-platform/. AstraZeneca Vivli member page is also available outlining further details: https://vivli.org/ourmember/astrazeneca/.

## Supplemental Material

Supplemental Table 1. Baseline characteristics of participants by early change in albuminuria at month 4 and by treatment status (placebo or dapagliflozin).

Supplemental Table 2. Baseline characteristics of participants by quartiles of early change in albuminuria at month 4 and by treatment status (placebo or dapagliflozin).

Supplemental Table 3. Association of early urinary albumin–creatinine ratio (UACR) change (%) at 4 months of using the assigned study treatment with primary and kidney composite end points in patients with and without type 2 diabetes.

Supplemental Table 4. Baseline characteristics of participants by residual level of albuminuria at month 4 and by treatment status (placebo or dapagliflozin).

Supplemental Figure 1. Risk of the primary and kidney end points by baseline and albuminuria level at month 4.

Supplemental Figure 2. Association of albuminuria at month 4 with primary and kidney end points in patients randomized to placebo or dapagliflozin, assessed by type 2 diabetes status.
