# Supplementary material for: Baseline, Early Changes, and Residual Albuminuria: Post Hoc Analysis of a Randomized Clinical Trial of Dapagliflozin in Chronic Kidney Disease
Source: Clin J Am Soc Nephrol. 2024 Dec 9;19(12):1574–84. doi: 10.2215/CJN.0000000000000550 (PMC11637702; doi:10.2215/CJN.0000000000000550)
Supplement: Supplementary file 1 [file cjasn-19-1574-s001.pdf]

ASN American Society of Nephrology | JASN Journal of the American Society of Nephrology | KIDNEY360 Accessing Our World From Every Angle | CJASN Clinical Journal of the American Society of Nephrology

ASN Journal Disclosure Form

As per ASN journal policy, I have disclosed any financial relationships or commitments I have held in the past 36 months as included below. I have listed my Current Employer below to indicate there is a relationship requiring disclosure. If no relationship exists, my Current Employer is not listed.

G. Chertow reports the following:
Employer: Stanford University School of Medicine; Consultancy: Akebia, Ardelyx, AstraZeneca, Beren, CalciMedica, Calico, Miromatrix, Panoramic, Sanifit, Toku, Unicycive, Vertex; Ownership Interest: Ardelyx, CloudCath, Durect, DxNow, Eliaz Therapeutics, Outset, Renibus, Unicycive; Research Funding: NIDDK, NIAID, CSL Behring; Advisory or Leadership Role: Board of Directors, Satellite Healthcare, Co-Editor, Brenner & Rector's The Kidney (Elsevier); and Other Interests or Relationships: DSMB service: NIDDK, Aethlon, Bayer, Mineralys, ReCor.

I understand that the information above will be published within the journal article, if accepted, and that failure to comply and/or to accurately and completely report the potential financial conflicts of interest could lead to the following: 1) Prior to publication, article rejection, or 2) Post-publication, sanctions ranging from, but not limited to, issuing a correction, reporting the inaccurate information to the authors' institution, banning authors from submitting work to ASN journals for varying lengths of time, and/or retraction of the published work.

Name: Glenn M Chertow
Manuscript ID: CJASN-2024-000410R1
Manuscript Title: Baseline, early changes, and residual albuminuria in a post-hoc analysis of a placebo-controlled-randomized trial of dapagliflozin in CKD
Date of Completion: July 23, 2024
Disclosure Updated Date: June 18, 2024

ASN American Society of Nephrology | JASN Journal of the American Society of Nephrology | KIDNEY360 Accessing Our World From Every Angle | CJASN Clinical Journal of the American Society of Nephrology

ASN Journal Disclosure Form

As per ASN journal policy, I have disclosed any financial relationships or commitments I have held in the past 36 months as included below. I have listed my Current Employer below to indicate there is a relationship requiring disclosure. If no relationship exists, my Current Employer is not listed.

R. Correa-Rotter reports the following:
Employer: Instituto Nacional de Ciencias Médicas y Nutrición Salvador Zubiran, MEXICO and; Universidad Nacional Autónoma de México, MEXICO; Consultancy: Astra Zeneca, Boheringer Ingelheim, Bayer, Chinook, Novonordisk; Research Funding: Astra Zeneca, Novonordisk, Roche, Chinook, GSK; Honoraria: Amgen, Astra Zeneca, Boehringer Ingelheim, Sanofi, Bayer, Amgen; Advisory or Leadership Role: Membership Steering Committee of DAPA CKD, Astra Zeneca.; National Leader ASCEND study, GSK; National Leader FLOW study, Novonordisk, membership steering Committee FINE-REAL, Bayer.. Editorial Board Nefrologia Latinoamericana, Revista de Investigación Clinica, American Journal of Kidney Diseases. y Associate Editor: Blood Purification Associate Editor. Member of the Steering Committee of the World Kidney Day organization.; Speakers Bureau: Amgen, Astra Zeneca, Boehringer Ingelheim, Abbvie, Sanofi, Bayer, Novonordisk; and Other Interests or Relationships: Member of ASN, of International Society of Nephrology; Member of National Kidney Foundation; Member Mexican Institute for Research in Nephrology; Member Latin American Society of Nephrology and Hypertension; Member EDTA/ERA.

I understand that the information above will be published within the journal article, if accepted, and that failure to comply and/or to accurately and completely report the potential financial conflicts of interest could lead to the following: 1) Prior to publication, article rejection, or 2) Post-publication, sanctions ranging from, but not limited to, issuing a correction, reporting the inaccurate information to the authors' institution, banning authors from submitting work to ASN journals for varying lengths of time, and/or retraction of the published work.

Name: Ricardo Correa-Rotter
Manuscript ID: CJASN-2024-000410R1
Manuscript Title: Baseline, early changes, and residual albuminuria in a post-hoc analysis of a placebo-controlled-randomized trial of dapagliflozin in CKD
Date of Completion: July 22, 2024
Disclosure Updated Date: May 21, 2024

ASN American Society of Nephrology | JASN Journal of the American Society of Nephrology | KIDNEY360 Accessing Our World From Every Angle | CJASN Clinical Journal of the American Society of Nephrology

ASN Journal Disclosure Form

As per ASN journal policy, I have disclosed any financial relationships or commitments I have held in the past 36 months as included below. I have listed my Current Employer below to indicate there is a relationship requiring disclosure. If no relationship exists, my Current Employer is not listed.

R. Gansevoort reports the following:
Employer: Univ Med Ctr Groningen; Consultancy: AstraZeneca, Bayer, Galapagos, Mironid, Sanofi-Genzyme,; Research Funding: AstraZeneca, Abbvie, Bayer, Galapagos, Otsuka Pharmaceuticals, Roche, Sanofi-Genzyme,; Honoraria: Galapagos, Otsuka Pharmaceuticals, Mironid.; and Advisory or Leadership Role: Editor of NDT, cJASN, Am J Kid Dis, J Nephrol, Nephron Clin Pract, Kidney 360. Member of the Council of the European Renal Association.

I understand that the information above will be published within the journal article, if accepted, and that failure to comply and/or to accurately and completely report the potential financial conflicts of interest could lead to the following: 1) Prior to publication, article rejection, or 2) Post-publication, sanctions ranging from, but not limited to, issuing a correction, reporting the inaccurate information to the authors' institution, banning authors from submitting work to ASN journals for varying lengths of time, and/or retraction of the published work.

Name: Ron T. Gansevoort
Manuscript ID: CJASN-2024-000410R1
Manuscript Title: Baseline, early changes, and residual albuminuria in a post-hoc analysis of a placebo-controlled-randomized trial of dapagliflozin in CKD
Date of Completion: July 23, 2024
Disclosure Updated Date: November 29, 2023

ASN American Society of Nephrology | JASN Journal of the American Society of Nephrology | KIDNEY360 Accessing Our World From Every Angle | CJASN Clinical Journal of the American Society of Nephrology

ASN Journal Disclosure Form

As per ASN journal policy, I have disclosed any financial relationships or commitments I have held in the past 36 months as included below. I have listed my Current Employer below to indicate there is a relationship requiring disclosure. If no relationship exists, my Current Employer is not listed.

H. Heerspink reports the following:
Employer: University Medical Center Groningen; Consultancy: Ongoing consultancy agreements with AstraZeneca, Alexion, Bayer, Boehringer Ingelheim, CSL Behring, Chinook, Dimerix, Eli-Lilly, Gilead, Janssen, Novartis, NovoNordisk, Travere Pharmaceuticals; Research Funding: AstraZeneca, Boehringer Ingelheim, NovoNordisk and Janssen research support (grant funding directed to employer); Honoraria: Lecture fees from AstraZeneca and NovoNordisk; and Speakers Bureau: AstraZeneca.

I understand that the information above will be published within the journal article, if accepted, and that failure to comply and/or to accurately and completely report the potential financial conflicts of interest could lead to the following: 1) Prior to publication, article rejection, or 2) Post-publication, sanctions ranging from, but not limited to, issuing a correction, reporting the inaccurate information to the authors' institution, banning authors from submitting work to ASN journals for varying lengths of time, and/or retraction of the published work.

Name: Hiddo Jan L. Heerspink
Manuscript ID: CJASN-2024-000420R1
Manuscript Title: Baseline, early changes, and residual albuminuria in a post-hoc analysis of a placebo-controlled-randomized trial of dapagliflozin in CKD
Date of Completion: July 24, 2024
Disclosure Updated Date: April 22, 2024

ASN
American Society of Nephrology
JASN
Journal of the American Society of Nephrology
KIDNEY360
Accessing Our World From Every Angle
CJASN
Clinical Journal of the American Society of Nephrology

ASN Journal Disclosure Form

As per ASN journal policy, I have disclosed any financial relationships or commitments I have held in the past 36 months as included below. I have listed my Current Employer below to indicate there is a relationship requiring disclosure. If no relationship exists, my Current Employer is not listed.

A. Langkilde reports the following:
Employer: AstraZeneca; and Ownership Interest: AstraZeneca.

I understand that the information above will be published within the journal article, if accepted, and that failure to comply and/or to accurately and completely report the potential financial conflicts of interest could lead to the following: 1) Prior to publication, article rejection, or 2) Post-publication, sanctions ranging from, but not limited to, issuing a correction, reporting the inaccurate information to the authors' institution, banning authors from submitting work to ASN journals for varying lengths of time, and/or retraction of the published work.

Name: Anna Maria Langkilde
Manuscript ID: CJASN-2024-000410R2
Manuscript Title: Baseline, Early Changes, and Residual Albuminuria: Post-hoc Analysis of a Clinical Trial of Dapagliflozin in Chronic Kidney Disease,
Date of Completion: October 9, 2024
Disclosure Updated Date: October 9, 2024

ASN American Society of Nephrology | JASN Journal of the American Society of Nephrology | KIDNEY360 Accessing Our World From Every Angle | CJASN Clinical Journal of the American Society of Nephrology

ASN Journal Disclosure Form

As per ASN journal policy, I have disclosed any financial relationships or commitments I have held in the past 36 months as included below. I have listed my Current Employer below to indicate there is a relationship requiring disclosure. If no relationship exists, my Current Employer is not listed.

P. Rossing reports the following:
Employer: Steno Diabetes Center Copenhagen; Research Funding: Novo Nordisk , AstraZeneca, Bayer; Honoraria: Boehringer Ingelheim, AstraZeneca, Abbott, Novo Nordisk, all honoraria to institution; and Advisory or Leadership Role: Astra Zeneca Bayer , novo nordisk, Gilead all honoraria to institution.

I understand that the information above will be published within the journal article, if accepted, and that failure to comply and/or to accurately and completely report the potential financial conflicts of interest could lead to the following: 1) Prior to publication, article rejection, or 2) Post-publication, sanctions ranging from, but not limited to, issuing a correction, reporting the inaccurate information to the authors' institution, banning authors from submitting work to ASN journals for varying lengths of time, and/or retraction of the published work.

Name: Peter Rossing
Manuscript ID: CJASN-2024-000410R1
Manuscript Title: Baseline, early changes, and residual albuminuria in a post-hoc analysis of a placebo-controlled-randomized trial of dapagliflozin in CKD
Date of Completion: July 22, 2024
Disclosure Updated Date: June 13, 2024

ASN American Society of Nephrology
JASN Journal of the American Society of Nephrology
KIDNEY360 Accessing Our World From Every Angle
CJASN Clinical Journal of the American Society of Nephrology

ASN Journal Disclosure Form

As per ASN journal policy, I have disclosed any financial relationships or commitments I have held in the past 36 months as included below. I have listed my Current Employer below to indicate there is a relationship requiring disclosure. If no relationship exists, my Current Employer is not listed.

D. Sjostrom reports the following:
Employer: AstraZeneca LP; and Ownership Interest: AstraZeneca LP.

I understand that the information above will be published within the journal article, if accepted, and that failure to comply and/or to accurately and completely report the potential financial conflicts of interest could lead to the following: 1) Prior to publication, article rejection, or 2) Post-publication, sanctions ranging from, but not limited to, issuing a correction, reporting the inaccurate information to the authors' institution, banning authors from submitting work to ASN journals for varying lengths of time, and/or retraction of the published work.

Name: David Sjostrom
Manuscript ID: CJASN-2024-000410R1
Manuscript Title: Baseline, early changes, and residual albuminuria in a post-hoc analysis of a placebo-controlled-randomized trial of dapagliflozin in CKD
Date of Completion: July 24, 2024
Disclosure Updated Date: May 16, 2024

ASN American Society of Nephrology | JASN Journal of the American Society of Nephrology | KIDNEY360 Accessing Our World From Every Angle | CJASN Clinical Journal of the American Society of Nephrology

ASN Journal Disclosure Form

As per ASN journal policy, I have disclosed any financial relationships or commitments I have held in the past 36 months as included below. I have listed my Current Employer below to indicate there is a relationship requiring disclosure. If no relationship exists, my Current Employer is not listed.

R. Toto reports the following:
Employer: University of Texas Southwestern Medical Center; Consultancy: Amgen, Astra-Zeneca, Boehringer-Ingelheim, Novartis, Alnylm Pharma; Research Funding: NIH; Vertex pharma; Honoraria: Amgen, Astra-Zeneca, Boehringer-Ingelheim, Novartis, Alnylm Pharma; and Advisory or Leadership Role: Astra-Zeneca, Boehringer-Ingelheim, Novartis, Novo Nordisk, CinCor, Calliditas, Medscape.

I understand that the information above will be published within the journal article, if accepted, and that failure to comply and/or to accurately and completely report the potential financial conflicts of interest could lead to the following: 1) Prior to publication, article rejection, or 2) Post-publication, sanctions ranging from, but not limited to, issuing a correction, reporting the inaccurate information to the authors' institution, banning authors from submitting work to ASN journals for varying lengths of time, and/or retraction of the published work.

Name: Robert D. Toto
Manuscript ID: CJASN-2024-000410R2
Manuscript Title: Baseline, Early Changes, and Residual Albuminuria: Post-hoc Analysis of a Clinical Trial of Dapagliflozin in Chronic Kidney Disease
Date of Completion: August 27, 2024
Disclosure Updated Date: August 27, 2024

ASN American Society of Nephrology · JASN Journal of the American Society of Nephrology · KIDNEY360 Accessing Our World From Every Angle · CJASN Clinical Journal of the American Society of Nephrology

ASN Journal Disclosure Form

As per ASN journal policy, I have disclosed any financial relationships or commitments I have held in the past 36 months as included below. I have listed my Current Employer below to indicate there is a relationship requiring disclosure. If no relationship exists, my Current Employer is not listed.

D. van Mil reports the following:
Employer: University Medical Center Groningen, University of Groningen

I understand that the information above will be published within the journal article, if accepted, and that failure to comply and/or to accurately and completely report the potential financial conflicts of interest could lead to the following: 1) Prior to publication, article rejection, or 2) Post-publication, sanctions ranging from, but not limited to, issuing a correction, reporting the inaccurate information to the authors' institution, banning authors from submitting work to ASN journals for varying lengths of time, and/or retraction of the published work.

Name: Dominique van Mil
Manuscript ID: CJASN-2024-000410R1
Manuscript Title: Baseline, early changes, and residual albuminuria in a post-hoc analysis of a placebo-controlled randomized trial of dapagliflozin in CKD
Date of Completion: July 3, 2024
Disclosure Updated Date: July 3, 2024

ASN
American Society of Nephrology
JASN
Journal of the American Society of Nephrology
KIDNEY360
Accessing Our World From Every Angle
CJASN
Clinical Journal of the American Society of Nephrology

ASN Journal Disclosure Form

As per ASN journal policy, I have disclosed any financial relationships or commitments I have held in the past 36 months as included below. I have listed my Current Employer below to indicate there is a relationship requiring disclosure. If no relationship exists, my Current Employer is not listed.

P. Vart reports the following:
Employer: University Medical Center Groningen; Ownership Interest: Apple, Tesla, MicroSoft, Nio, Airbus, Boeing; Research Funding: AstraZeneca; and Advisory or Leadership Role: Editor Nephrology Dialysis Transplantation (unpaid); Editor Scientific Reports (unpaid).

I understand that the information above will be published within the journal article, if accepted, and that failure to comply and/or to accurately and completely report the potential financial conflicts of interest could lead to the following: 1) Prior to publication, article rejection, or 2) Post-publication, sanctions ranging from, but not limited to, issuing a correction, reporting the inaccurate information to the authors' institution, banning authors from submitting work to ASN journals for varying lengths of time, and/or retraction of the published work.

Name: Priya Vart
Manuscript ID: CJASN-2024-000410R1
Manuscript Title: Baseline, early changes, and residual albuminuria in a post-hoc analysis of a placebo-controlled-randomized trial of dapagliflozin in CKD
Date of Completion: August 1, 2024
Disclosure Updated Date: July 5, 2024

ASN American Society of Nephrology | JASN Journal of the American Society of Nephrology | KIDNEY360 Accessing Our World From Every Angle | CJASN Clinical Journal of the American Society of Nephrology

ASN Journal Disclosure Form

As per ASN journal policy, I have disclosed any financial relationships or commitments I have held in the past 36 months as included below. I have listed my Current Employer below to indicate there is a relationship requiring disclosure. If no relationship exists, my Current Employer is not listed.

D. Wheeler reports the following:
Employer: University College London; Consultancy: Fees from: Astellas, AstraZeneca, Bayer, Boehringer Ingelheim, Eledon, Galderma, George Clinical, Gilead, GlaxoSmithKline, Janssen, Merck Sharp and Dohme, Mineralys, Pathalys, Pfizer, ProKidney, Sana, Tricida, Vifor, Zydus for Talks, Advisory Boards, Trial Committees and Consultancy; Research Funding: National Institute for Health Research; Honoraria: Amgen, Astellas, AstraZeneca, Bayer, Boehringer Ingelhiem, GalaxoSmithKline, Janssen, Napp, Mundipharma, Merck Sharp and Dohme, Napp, Reata, Pharmacosmos, Vifor Fresenius.; Advisory or Leadership Role: National Institute of Health Research Clinical Lead for Renal Disorders.; and Speakers Bureau: Amgen, AstraZeneca, Astellas, Janssen, Mundipharma, Napp, Merck Sharp and Dohme, Vifor Fresenius.

I understand that the information above will be published within the journal article, if accepted, and that failure to comply and/or to accurately and completely report the potential financial conflicts of interest could lead to the following: 1) Prior to publication, article rejection, or 2) Post-publication, sanctions ranging from, but not limited to, issuing a correction, reporting the inaccurate information to the authors' institution, banning authors from submitting work to ASN journals for varying lengths of time, and/or retraction of the published work.

Name: David C. Wheeler
Manuscript ID: CJASN-2024-000410R1
Manuscript Title: Baseline, early changes, and residual albuminuria in a post-hoc analysis of a placebo-controlled-randomized trial of dapagliflozin in CKD
Date of Completion: July 23, 2024
Disclosure Updated Date: May 17, 2024
