# Supplementary material for: Baseline, Early Changes, and Residual Albuminuria: Post Hoc Analysis of a Randomized Clinical Trial of Dapagliflozin in Chronic Kidney Disease
Source: Clin J Am Soc Nephrol. 2024 Dec 9;19(12):1574–84. doi: 10.2215/CJN.0000000000000550 (PMC11637702; doi:10.2215/CJN.0000000000000550)
Supplement: Supplementary file 2 [file cjasn-19-1574-s002.pdf]

Clinical Journal of the American Society of Nephrology - LICENSE TO PUBLISH

This LICENSE TO PUBLISH (this "License"), dated as of:

03 July 2024

DATE

(the "Effective Date"), is executed by the corresponding author listed on Schedule A (the "Author") to grant a license to the Medical Research Division of Wolters Kluwer Health, Inc., a Delaware corporation, having its principal place of business at Two Commerce Square, 2001 Market Street, Philadelphia, PA 19103
(the "Publisher", and together with the Author, each, a "Party", and together, the "Parties").

1. Grant of License

The Author hereby grants to the Publisher and its affiliates the exclusive, worldwide, royalty free, perpetual (for the duration of the applicable copyright) right and license to use the Work (as defined on Schedule A) for all commercial or educational purposes, including, but not limited to, publishing, reproducing, marketing, distributing (themselves and through distributors), sublicensing, and selling copies of the Work throughout the world for the Term. If the Author is a United States government employee, such license grant shall be limited to the extent the Author is able to grant such license.

2. Warranties, Indemnification, and Limitation of Liability

a. The Author represents and warrants to the Publisher that:

(i) the Author has the right and power to enter into this License, to grant the rights and licenses granted pursuant to this License, and to perform all of the Author's other obligations contained in this License;

(ii) the Author has not previously assigned, transferred or otherwise encumbered the rights or licenses granted pursuant to this License;

(iii) the person executing this License on the Author's behalf is authorized to do so;

(iv) the Work and the licenses granted herein do not and will not infringe upon, violate or misappropriate any intellectual property rights or any other proprietary right, contract or other right or interest of any third party;

(v) if the Work is a multi-authored Work, the Author has obtained written permission from each author of the Work to enter into this License on behalf such author, and each such author has read, understands and has agreed to the terms of this License;

(vi) the Author has obtained any necessary releases and permissions to quote from other sources in the Work and to include any works and materials in the Work, including all releases from patients whose names or likenesses are submitted as part of the Work; all such releases and permissions are in full force and effect, and the Author will promptly provide any such release or permission to the Publisher upon request by the Publisher;

(vii) neither the Work nor any content contained in the Work, in whole or in part, has been published or is being considered for publication other than in the Journal (as defined in Section 3.a.);

(viii) the Author has disclosed to the Publisher, prior to or simultaneously with submission of the Work, all intellectual contributions, technical help, financial or material support, and all financial or other relationships that may constitute or lead to a conflict of interest;

(vii) the Work is not subject to any rights of copyright other than the copyright of the Author and each other author of the Work;

(ix) the Work does not and will not violate the publicity or privacy rights of any third party, or libel or slander any third party;

(x) the Work does not and will not contain any scandalous, obscene, or negligently prepared information;

(xi) the Work is not and will not be fraudulent, plagiarized, or incorrectly attributed;

(xii) no aspect of the Author's personal or professional circumstances currently, or in the past thirty-six (36) months, causes the Author to have a conflict of interest with respect to the Work;

(xiii) neither the Author, nor any member of the Author's immediate family, nor any individual or entity with whom or which the Author has or has had a significant working relationship has received anything of value from a commercial

party related directly or indirectly to the subject of the Work;

(xiv) the Author has read and understands the statements on Schedule B and has completed Schedule B in its entirety; and

(xv) : If any author resides in Iran, Cuba, Syria, North Korea, Crimea, Russia, Belarus or the Donetsk People's Republic (DNR) or Luhansk People's Republic (LNR) regions of Ukraine, the article or Content, as applicable, such author represents and warrants that the Work has been prepared in a personal, academic or research capacity and not as an official representative or otherwise on behalf of the relevant government.

b. The Author hereby indemnifies the Publisher and its directors, officers, employees, agents, and representatives and agrees to defend and hold them harmless from and against any and all liability, damage, loss, costs or expenses (including reasonable attorney's fees and costs of settlement) incurred by any such party arising out of, or relating to any misrepresentation in, or breach or alleged breach of the Author's representations or warranties in this License. If the Author fails to promptly or diligently pursue any defense of any indemnified party, the indemnified parties, or any of them, may assume such defense at the Author's expense. The obligations of this indemnification will survive any termination or expiration of this License.

3. Creative Commons License

The Author acknowledges and agrees that the Work will be published by the Publisher in *JOURNAL* (the "Journal") and made freely available to users under the terms of the Creative Commons Licenses, which license will be selected by the Author pursuant to Section B:

(i) Creative Commons Attribution-NonCommercial-NoDerivatives 4.0 International Public License, as currently displayed at http://creativecommons.org/licenses/by-nc-nd/4.0/legalcode (the "CC BY-NC-ND"). The Author acknowledges and agrees that the Publisher is the exclusive "Licensor", as defined in the CC BY-NC- ND, of the Work and that the Publisher may make the Work freely available to all users under the terms of the CC BY-NC-ND.

(ii) Creative Commons Attribution 4.0 International Public License, as currently displayed at http://creativecommons.org/licenses/by/4.0/legalcode (the "CC BY"). The Author acknowledges and agrees that the Publisher is the exclusive "Licensor", as defined in the CC BY, of the Work and that the Publisher may make the Work freely available to all users under the terms of the CC BY.

4. Royalties

The Author acknowledges and agrees that this License entitles the Author to no royalties or fees. To the maximum extent permitted by law, the Author waives any and all rights the Author may have to collect royalties or other fees in relation to the Work or in respect of any use of the Work by the Publisher or its sublicensees.

5. Miscellaneous

a. Assignment. This License may not be assigned or transferred, in whole or in part, by the Author. The Publisher may freely assign this License. This License will be binding upon and inure to the benefit of the Parties hereto and their respective successors and permitted assigns.

b. Execution. Facsimile or Portable Document Format (PDF) signatures will be deemed original signatures for purposes of this License.

c. Entire Agreement; Amendment. This License sets forth the entire agreement of the Parties on the subject hereof and supersedes all previous or contemporaneous oral or written representations or agreements relating to the rights and duties provided herein, and may not be modified or amended except by written agreement of the Publisher.

d. Governing Law. This License shall be governed in all respects according to the laws of the State of New York without giving effect to the principles of conflict of law thereof.

e. Headings. All headings are for reference purposes only and shall not affect the meaning or interpretation of any provision hereof.

g. Severability. If any provision of this License is held to be illegal, invalid, or unenforceable under the present or future laws, then such provision shall be revised by a court of competent jurisdiction to be enforceable if permitted under applicable law, and otherwise shall be fully severable. In any event, this License shall be construed and enforced as if such illegal, invalid, or unenforceable provision had never comprised a part of this License, and the remaining provisions of this License shall remain in full force and effect and shall not be affected by the illegal, invalid, or unenforceable provision or by its severance from this License.

h. Status of the Parties. The Parties are independent contractors. Nothing in this License is intended to or shall be construed to constitute or establish any agency, joint venture, partnership or fiduciary relationship between the Parties, and neither Party has the right or authority to bind the other Party nor shall either Party be responsible for the acts or omissions of the other Party.

i. Waiver; Amendment. The waiver by the Publisher of or the failure by the Publisher to claim a breach of any provision of this License shall not be, or be held to be, a waiver of any subsequent breach or affect in any way the further effectiveness of any such provision. No term or condition of this License may be waived except by an agreement by the Publisher in writing.

j. Waiver of Jury Trial. THE AUTHOR WAIVES THE AUTHOR'S RIGHT TO A JURY TRIAL IN CONNECTION WITH ANY DISPUTE

OR LEGAL PROCEEDING ARISING OUT OF THIS AGREEMENT
OR THE SUBJECT MATTER HEREOF.

SCHEDULE A to License to Publish

The Author must complete this Schedule A in its entirety. The Publisher is unable to publish the Work unless this Schedule A is completed.

Baseline, early changes, and residual albumiuria in a post-hoc analysis of a placebo-controlled randomized trial of dapagliflozin in CKD

Article Title (the "Work")

Hiddo J.L. Heerspink

Corresponding Author Name (the "Author")

Hiddo J.L. Heerspink

Copyright Owner's Name

CJASN-2024-000410R1

Manuscript Number (Optional)

SCHEDULE B to License to Publish

☐ **GOVERNMENT EMPLOYEES**

If the Work or a portion of it has been created in the course of any author's employment by the United States Government, check the "Government" box at the end of this form. A work prepared by a government employee as part of his or her official duties is called a "work of the U.S. Government" and is not subject to copyright. If it is not prepared as part of the employee's official duties, it may be subject to copyright.

If “Government” is chosen, please skip to page 7 and do not choose a Creative Commons License. The work will be published with “Written work prepared by employees of the Federal Government as part of their official duties is, under the U.S. Copyright Act, a “work of the United States Government” for which copyright protection under Title 17 of the United States Code is not available. As such, copyright does not extend to the contributions of employees of the Federal Government.”

CREATIVE COMMONS LICENSE

Please select the Creative Commons License pursuant to which the Publisher will license the Work (a description of each license, and the license terms, can be reviewed at http://www.creativecommons.org/licenses):

Select one from the list below:

☑ **Attribution-NonCommercial-NoDerivs CC BY-NC-ND.** Creative Commons describes this license as follows: "This license is the most restrictive of our six main licenses, only allowing others to download your works and share them with others as long as they credit you, but they can't change them in any way or use them commercially."

☐ **Attribution CC BY**. Creative Commons describes this license as follows: "This license lets others distribute, remix, tweak, and build upon your work, even commercially, as long as they credit you for the original creation. This is the most accommodating of licenses offered. Recommended for maximum dissemination and use of licensed materials."

SIGNATURE PAGE – License to Publish

IN WITNESS WHEREOF, the Author has executed this License, effective as of the Effective Date.

Hiddo J.L. Heerspink

PRINT NAME

SIGNATURE

Important Note: Once you electronically sign this form, you will not be able to make any additional changes to it.

To electronically sign this form, click the signature field above and provide the information requested in the dialog boxes.
