# Supplementary material for: Baseline, Early Changes, and Residual Albuminuria: Post Hoc Analysis of a Randomized Clinical Trial of Dapagliflozin in Chronic Kidney Disease
Source: Clin J Am Soc Nephrol. 2024 Dec 9;19(12):1574–84. doi: 10.2215/CJN.0000000000000550 (PMC11637702; doi:10.2215/CJN.0000000000000550)

Supplementary Material

Supplementary Table 1. Baseline characteristics of participants by early change in albuminuria at month 4 and by treatment status (placebo or dapagliflozin) 2

Supplementary Table 2. Baseline characteristics of participants by quartiles of early change in albuminuria at month 4 and by treatment status (placebo or dapagliflozin) 4

Supplementary Table 3. Association of early urinary albumin–creatinine ratio (UACR) change (%) at 4 months of using the assigned study treatment with primary and kidney composite end points in patients with and without type 2 diabetes 6

Supplementary Table 4. Baseline characteristics of participants by residual level of albuminuria at month 4 and by treatment status (placebo or dapagliflozin) 7

Supplementary Figure 1. Risk of the primary and kidney end point by baseline and albuminuria level at month 4 9

Supplementary Figure 2. Association of albuminuria at month 4 with primary and kidney end points in patients randomized to placebo or dapagliflozin, assessed by type 2 diabetes status 10

Supplementary Table 1. Baseline characteristics of participants by early change in albuminuria at month 4 and by treatment status (placebo or dapagliflozin)

	Early change in albuminuria at month 4							
	>30% decrease		0% to ≤30% decrease		0 to <30% increase		≥30% increase	
**Characteristics**	Dapagliflozin	Placebo	Dapagliflozin	Placebo	Dapagliflozin	Placebo	Dapagliflozin	Placebo
n (%)	1187 (30.1)	726 (18.4)	332 (8.4)	431 (10.9)	236 (6.0)	381 (9.7)	223 (5.7)	424 (10.8)
UACR change in % at Month 4, median (IQR)	-79.1 (-122.7 to 53.5)	-71.8 (-111.1 to -46.6)	-16.5 (-23.3 to -8.3)	-14.5 (-22.1 to 6.8)	12.2 (4.9 to 20.9)	13.8 (6.6 to 21.5)	57.5 (50.0 to 90.4)	59.1 (42.2 to 82.5)
Baseline UACR, mg/g, median (IQR)	980 (466-1856	898.5 (462-1717)	1070 (512-2067	1038 (523 to 2166)	1056 (493-2469)	1044 (478-2157)	669 (373-1290)	836 (459-1533)
UACR baseline category, mg/g, n %								
<300	134 (11.3)	72 (9.9)	29 (8.7)	33 (7.7)	20 (8.5)	35 (9.2)	41 (18.4)	51 (12.0)
≥300–<1000	468 (39.4)	319 (43.9)	129 (38.9)	173 (40.1)	94 (39.8)	148 (38.9)	109 (48.9)	196 (46.2)
≥1000–<3000	463 (39.0)	272 (37.5)	136 (41.0)	166 (38.5)	81 (34.3)	138 (36.2)	55 (24.7)	149 (35.1)
≥3000	122 (10.3)	63 (8.7)	38 (11.5)	59 (13.8)	41 (17.4)	60 (15.8)	18 (8.1)	28 (6.6)
Age, years	63.0 (11.3)	63.6 (11.2)	61.4 (12.5)	62.3 (12.0)	60.1 (12.9)	60.7 (13.1)	60.8 (12.2)	60.6 (12.4)
Men, n (%)	773 (62.1)	455 (62.7)	332 (71.2)	317 (73.6)	165 (69.9)	260 (68.2)	150 (67.3)	283 (66.8)
Race, n (%)								
Asian	334 (28.1)	185 (25.6)	124 (37.4)	131 (30.4)	80 (33.9)	117 (30.7)	83 (37.2)	156 (36.8)
Black/African American	72 (6.1)	31 (4.3)	13 (3.9)	14 (3.3)	8 (3.4)	16 (4.2)	6 (2.7)	20 (4.7)
Other	104 (8.8)	74 (10.2)	26 (7.8)	42 (9.7)	22 (9.3)	25 (6.6)	14 (6.3)	30 (7.1)
White	677 (57.0)	436 (60.1)	169 (50.9)	244 (56.6)	126 (53.4)	223 (58.5)	120 (53.8)	218 (51.4)
Current smoker, n (%)	140 (11.8)	92 (12.7)	43 (13.0)	70 (16.2)	34 (14.4)	57 (15.0)	32 (14.4)	49 (11.6)
CKD diagnosis, n (%)								
Diabetic nephropathy	750 (63.2)	418 (57.6)	189 (56.9)	251 (58.2)	127 (53.8)	219 (57.5)	133 (59.6)	247 (58.3)
Hypertensive CKD	173 (14.6)	141 (19.4)	58 (17.5)	71 (16.5)	43 (18.2)	64 (16.8)	29 (13.0)	67 (15.8)
Glomerulonephritis	144 (12.1)	91 (12.5)	60 (18.1)	71 (16.5)	49 (20.8)	66 (17.3)	38 (17.0)	80 (18.9)
Other or unknown	120 (10.1)	76 (10.5)	25 (7.5)	38 (8.8)	17 (7.2)	32 (8.4)	23 (10.3)	30 (7.1)
History of diabetes, n (%)	850 (71.6)	489 (67.4)	215 (64.8)	303 (70.3)	146 (61.9)	248 (65.1)	154 (69.0)	292 (68.9)
Duration of diabetes, years, median (IQR)*	14.7 (7.6-21.5)	12.9 (6.8-20.6)	13.8 (7.6-20.4)	14.3 (7.7-20.7)	13.2 (6.1-19.9)	15.7 (8.5-21.2)	11.0 (5.1-20.2)	14.1 (7.7-20.2)
History of cardiovascular disease, n (%)	479 (40.4)	296 (40.8)	132 (39.8)	141 (32.7)	72 (30.5)	137 (36.0)	86 (38.6)	161 (38.0)

History of heart failure, n (%)		137 (11.5)	106 (14.6)		39 (11.8)	33 (7.7)		18 (7.6)	32 (8.4)		34 (15.3)	47 (11.1)
Weight, kg		82.7 (20.8)	81.9 (20.0)		80.9 (18.4)	84.6 (21.6)		81.6 (20.7)	83.5 (22.0)		80.7 (19.9)	82.1 (21.6)
Body mass index, kg/m^2		29.9 (6.2)	29.9 (6.1)		28.9 (5.6)	30.0 (6.1)		29.3 (6.3)	30.0 (6.9)		29.3 (5.5)	29.7 (6.2)
Systolic blood pressure, mmHg		138.3 (17.4)	140.8 (18.1)		136.7 (16.8)	137.8 (17.0)		135.2 (18.5)	135.9 (17.0)		132.9 (16.6)	133.7 (15.6)
Diastolic blood pressure, mmHg		77.1 (10.4)	78.6 (10.5)		78.4 (11.1)	77.1 (9.9)		77.7 (10.4)	77.5 (10.4)		77.3 (10.6)	76.1 (9.6)
HbA1c, %		7.2 (1.7)	7.0 (1.6)		7.0 (1.7)	7.1 (1.7)		7.0 (1.9)	7.0 (1.8)		7.1 (1.8)	7.3 (2.0)
eGFR, ml/min per 1.73 m^2		43.8 (12.3)	44.2 (12.3)		42.1 (12.7)	43.0 (12.7)		43.4 (13.0)	42.2 (12.1)		43.5 (12.1)	42.8 (12.9)
Hemoglobin, g/L		128.6 (17.4)	128.9 (18.0)		129.0 (18.7)	129.4 (17.9)		128.6 (19.3)	127.0 (18.0)		127.8 (19.4)	125.5 (17.4)
Baseline ACE inhibitor/ARB use, n (%)		1169 (98.5)	719 (99.0)		327 (98.5)	423 (98.1)		235 (99.6)	366 (96.1)		218 (97.8)	412 (97.2)
Baseline diuretic use, n (%)		563 (47.4)	331 (45.6)		124 (37.4)	188 (43.6)		102 (43.2)	170 (44.6)		97 (43.5)	198 (46.7)

Data are presented mean (SD), unless otherwise indicated. ACE, angiotensin-converting enzyme; ARB, angiotensin-receptor blocker; CKD, chronic kidney disease; IQR, interquartile range; n, number; SD, standard deviation; UACR, urinary albumin–creatinine ratio. * Among those participants with a history of diabetes.

Supplementary Table 2. Baseline characteristics of participants by quartiles of early change in albuminuria at month 4 and by treatment status (placebo or dapagliflozin)

	Early change in albuminuria at month 4							
	Quartile 1		Quartile 2		Quartile 3		Quartile 4	
**Characteristics**	Dapagliflozin	Placebo	Dapagliflozin	Placebo	Dapagliflozin	Placebo	Dapagliflozin	Placebo
n (%)	637 (16.2)	348 (8.8)	579 (14.7)	406 (10.3)	419 (10.6)	566 (14.4)	343 (8.7)	642 (16.3)
UACR change in % at Month 4, median (IQR)	-118.1 (157.8 to -98.9)	-114.3 (-168.6 to -91.9)	-50.4 (-62.0 to -38.2)	-46.0 (-58.9 to -36.6)	-9.1 (-19.2 to 0.8)	-7.7 (-18.0 to 1.9)	40.1 (24.5 to 68.4)	42.0 (24.5 to 70.0)
Baseline UACR, mg/g, median (IQR)	975 (474-1836)	793 (437-1627)	1024 (465-1890)	1001 (521-1900)	1115 (507-2247)	1005 (489-2316)	730 (406-1497)	904 (478-1766)
UACR baseline category, mg/g, n %								
<300	69 (10.8)	40 (11.5)	67 (11.6)	34 (8.4)	37 (8.8)	47 (8.3)	51 (14.9)	70 (10.9)
≥300–<1000	258 (40.5)	159 (45.7)	219 (37.8)	169 (41.6)	156 (37.2)	232 (41.0)	167 (48.7)	276 (43.0)
≥1000–<3000	239 (37.5)	126 (36.2)	236 (40.8)	158 (38.9)	170 (40.6)	205 (36.2)	90 (26.2)	236 (36.8)
≥3000	71 (11.2)	23 (6.6)	57 (9.8)	45 (11.1)	56 (13.4)	82 (14.5)	35 (10.2)	60 (9.4)
Age, years	63.3 (11.0)	64.1 (11.2)	62.6 (11.7)	62.9 (11.2)	31.3 (12.9)	61.4 (12.4)	60.3 (12.4)	61.0 (12.6)
Men, n (%)	392 (61.5)	199 (57.2)	402 (69.4)	273 (67.2)	304 (72.6)	408 (72.1)	226 (65.9)	435 (67.8)
Race, n (%)								
Asian	171 (26.8)	94 (27.0)	173 (29.9)	103 (25.4)	150 (35.8)	166 (29.3)	127 (37.0)	226 (35.2)
Black/African American	34 (5.3)	17 (4.9)	38 (6.6)	14 (3.5)	17 (4.1)	22 (3.9)	10 (2.9)	28 (4.4)
Other	68 (10.7)	25 (7.2)	36 (6.2)	53 (13.1)	35 (8.4)	49 (8.7)	27 (7.9)	44 (6.9)
White	364 (57.1)	212 (60.9)	332 (57.3)	236 (58.1)	217 (51.8)	329 (58.1)	179 (52.2)	344 (53.6)
Current smoker, n (%)	59 (9.3)	43 (12.4)	86 (14.9)	53 (13.1)	56 (13.4)	329 (58.1)	48 (14.0)	344 (53.6)
CKD diagnosis, n (%)								
Diabetic nephropathy	435 (68.3)	190 (54.6)	335 (57.9)	244 (60.1)	235 (56.1)	329 (58.1)	194 (56.6)	372 (57.9)
Hypertensive CKD	76 (11.9)	79 (22.7)	101 (17.4)	67 (16.5)	76 (18.1)	90 (15.9)	64 (18.7)	107 (16.7)
Glomerulonephritis	66 (10.4)	31 (8.9)	82 (14.2)	65 (16.0)	79 (18.9)	94 (16.6)	50 (14.6)	118 (18.4)
Other or unknown	60 (9.4)	48 (13.8)	61 (10.5)	30 (7.4)	29 (6.9)	53 (9.4)	35 (10.2)	45 (7.0)
History of diabetes, n (%)	487 (76.5)	228 (65.5)	385 (66.5)	280 (69.0)	269 (64.2)	386 (68.2)	224 (65.3)	438 (68.2)
Duration of diabetes, years, median (IQR)*	15.1 (8.1-22.3)	12.0 (6.0-20.3)	13.6 (7.1-20.5)	14.1 (7.7-21.0)	13.1 (6.9-20.0)	14.1 (7.9-20.4)	12.6 (5.9-20.5)	15.1 (8.1-21.0)
History of cardiovascular disease, n (%)	279 (43.8)	155 (44.5)	208 (35.9)	151 (37.2)	167 (39.9)	192 (33.9)	115 (33.5)	327 (36.9)

History of heart failure, n (%)		88 (13.8)	73 (21.0)		51 (8.8)	36 (8.9)		51 (12.2)	46 (8.1)		38 (11.2)	63 (9.8)
Weight, kg		81.8 (20.8)	80.2 (19.0)		83.7 (20.5)	83.4 (20.8)		81.2 (19.0)	84.1 (21.6)		80.9 (20.3)	82.8 (21.9)
Body mass index, kg/m^2		29.8 (6.2)	29.7 (6.1)		29.9 (6.1)	30.1 (6.2)		29.0 (5.9)	30.0 (6.3)		29.4 (5.8)	29.8 (6.5)
Systolic blood pressure, mmHg		138.8 (17.4)	141.1 (17.8)		137.8 (17.3)	140.0 (18.4)		135.8 (17.2)	137.2 (16.9)		134.0 (17.5)	134.6 (16.2)
Diastolic blood pressure, mmHg		76.6 (10.2)	79.2 (10.7)		77.8 (10.6)	77.8 (10.2)		78.0 (10.9)	77.5 (10.2)		77.6 (10.7)	76.5 (9.8)
HbA1c, %		7.4 (1.8)	6.9 (1.5)		7.0 (1.8)	7.0 (1.6)		7.0 (1.8)	7.1 (1.7)		7.0 (1.7)	7.2 (1.9)
eGFR, ml/min per 1.73m^2		44.2 (12.0)	44.2 (12.0)		43.3 (12.6)	44.3 (12.5)		41.9 (12.6)	43.1 (12.6)		44.1 (12.6)	42.2 (12.6)
Hemoglobin, g/L		127.7 (17.1)	128.6 (18.3)		129.7 (17.5)	129.1 (17.7)		128.5 (19.1)	129.2 (18.1)		128.4 (19.3)	125.7 (17.4)
Baseline ACE inhibitor/ARB use, n (%)		626 (98.3)	344 (99.0)		572 (98.8)	403 (99.3)		413 (98.6)	550 (97.2)		338 (98.5)	623 (97.0)
Baseline diuretic use, n (%)		315 (49.5)	151 (44.3)		259 (44.7)	189 (46.6)		169 (40.3)	253 (44.7)		143 (41.7)	291 (45.3)

Data are presented mean (SD), unless otherwise indicated. ACE, angiotensin-converting enzyme; ARB, angiotensin-receptor blocker; CKD, chronic kidney disease; IQR, interquartile range; n, number; SD, standard deviation; UACR, urinary albumin–creatinine ratio. * Among those participants with a history of diabetes.

Supplementary Table 3. Association of early urinary albumin–creatinine ratio (UACR) change (%) at 4 months of using the assigned study treatment with primary and kidney composite end points in patients with and without type 2 diabetes

	**Quartile of UACR change (%)**			
**Primary end point**	**Quartile 1**<br>-119 (-165, -93)	**Quartile 2**<br>-49 (-61, -38)	**Quartile 3**<br>-8 (-19, 0.8)	**Quartile**<br>42 (27, 70)
With diabetes, N	715	665	655	662
Events, n (%)	61 (8.5)	70 (10.5)	90 (13.7)	125 (18.9)
HR (95% CI)	Ref.	1.11 (0.78, 1.58)	1.28 (0.91, 1.79)	2.21 (1.60, 3.07)
Without diabetes, N	270	320	330	323
Events, n (%)	12 (4.4)	23 (7.2)	35 (10.6)	50 (15.5)
HR (95% CI)	Ref.	1.67 (0.82, 3.39)	2.01 (1.03, 3.92)	3.34 (1.74, 6.41)
**Kidney end point**	**Quartile 1**<br>-111 (-153, -91)	**Quartile 2**<br>-49 (-62, -37)	**Quartile 3**<br>-8 (-18, 2)	**Quartile 4**<br>39 (22, 68)
With diabetes, N	715	665	655	662
Events, n (%)	39 (715)	50 (665)	70 (655)	106 (662)
HR (95% CI)	Ref.	1.14 (0.75, 1.75)	1.38 (0.92, 2.08)	2.88 (1.95, 4.26)
Without diabetes, N	270	320	330	323
Events, n (%)	7 (270)	21 (320)	28 (330)	48 (323)
HR (95% CI)	Ref.	2.38 (1.00, 5.68)	2.42 (1.04, 5.64)	5.10 (2.26, 1.53)

CI, confidence interval; HR, hazard ratio; UACR, urinary albumin–creatinine ratio.

Supplementary Table 4. Baseline characteristics of participants by residual level of albuminuria at month 4 and by treatment status (placebo or dapagliflozin)

	Residual level of albuminuria at Month 4							
	<500 (mg/g)		≥500 - <1000 (mg/g)		≥1000 - <2000 (mg/g)		≥2000 (mg/g)	
**Characteristics**	Dapagliflozin	Placebo	Dapagliflozin	Placebo	Dapagliflozin	Placebo	Dapagliflozin	Placebo
n (%)	881 (20.6)	682 (17.3)	434 (11.0)	428 (10.9)	385 (97.8)	414 (10.5)	278 (7.1)	436 (11.1)
UACR change in % at Month 4, median (IQR)	-79.0 (-133.0 to -40.3)	-53.2 (-107.5 to -13.7)	-37.8 (-73.6 to -0.7)	-13.5 (-50.9 to 19.8)	-27.0 (-56.9 to 11.3)	3.5 (-26.3 to 41.9)	4.7 (-20.1 to 32.6)	21.6 (-6.0 to 51.9)
Baseline UACR, mg/g, median (IQR)	466 (307-760)	429 (302-646)	1064 (725-1521)	802 (558-1259)	1809 (1216-2471)	1320 (930-1849)	3053 (2153-3997)	2801 (1871-3573)
UACR baseline category, mg/g, n %								
<300	207 (23.5)	169 (24.8)	13 (3.0)	18 (4.2)	3 (0.8)	3 (0.7)	1 (0.4)	1 (0.2)
≥300–<1000	536 (60.8)	446 (64.4)	183 (42.2)	250 (58.4)	67 (17.4)	114 (27.5)	14 (5.0)	26 (5.9)
≥1000–<3000	134 (15.2)	63 (9.2)	220 (50.7)	152 (35.5)	261 (67.8)	283 (68.4)	120 (43.1)	277 (51.8)
≥3000	4 (0.5)	4 (0.6)	18 (4.2)	8 (1.9)	54 (14.0)	14 (3.4)	143 (51.4)	184 (42.0)
Age, years	63.8 (11.5)	64.0 (11.3)	61.8 (12.2)	62.2 (12.0)	61.1 (11.7)	61.0 (13.0)	59.5 (11.9)	59.8 (12.1)
Men, n (%)	578 (65.6)	444 (65.1)	283 (65.2)	284 (66.4)	271 (70.4)	289 (69.8)	192 (69.1)	298 (68.0)
Race, n (%)								
Asian	252 (28.6)	187 (27.4)	145 (33.4)	128 (29.9)	124 (32.2)	128 (30.9)	100 (36.0)	146 (33.3)
Black/African American	49 (5.6)	28 (4.1)	17 (3.9)	21 (4.9)	17 (4.4)	17 (4.1)	16 (5.7)	15 (3.4)
Other	56 (6.4)	43 (6.3)	42 (9.7)	32 (7.5)	36 (9.4)	37 (8.9)	32 (11.5)	59 (13.5)
White	524 (59.5)	424 (62.3)	230 (53.0)	247 (57.7)	208 (54.0)	432 (56.0)	130 (46.8)	218 (49.8)
Current smoker, n (%)	97 (11.0)	79 (11.6)	48 (11.1)	58 (13.6)	54 (14.0)	59 (14.3)	50 (18.0)	72 (16.4)
CKD diagnosis, n (%)								
Diabetic nephropathy	536 (60.7)	382 (56.0)	238 (54.8)	228 (53.3)	228 (59.2)	233 (56.3)	198 (71.2)	292 (66.7)
Hypertensive CKD	145 (16.5)	153 (22.4)	71 (16.4)	86 (20.1)	61 (15.8)	59 (14.3)	26 (14.4)	45 (10.3)
Glomerulonephritis	104 (11.8)	80 (11.7)	86 (19.8)	71 (16.6)	61 (15.8)	79 (19.1)	40 (14.4)	78 (17.8)
Other or unknown	97 (11.0)	153 (22.4)	39 (9.0)	43 (10.1)	35 (9.1)	43 (10.4)	14 (5.0)	23 (5.3)
History of diabetes, n (%)	616 (69.9)	456 (66.9)	270 (62.2)	270 (63.1)	265 (68.8)	278 (67.2)	214 (77.0)	328 (74.9)
Duration of diabetes, years, median (IQR)*	13.0 (7.3-20.7)	13.0 (7.0-20.2)	15.0 (7.0-22.0)	13.5 (7.6-20.1)	14.9 (7.6-20.5)	14.7 (7.4-21.5)	13.5 (6.4-20.8)	16 (8.4-22.2)
History of cardiovascular disease, n (%)	360 (40.9)	275 (40.3)	151 (34.8)	153 (35.8)	145 (37.7)	148 (35.8)	113 (40.7)	159 (36.3)

History of heart failure, n (%)		101 (11.5)	94 (13.8)		46 (10.6)	48 (11.2)		43 (11.2)	40 (9.7)		38 (13.7)	36 (8)
Weight, kg		82.7 (20.8)	82.4 (20.4)		80.8 (19.1)	84.1 (21.7)		82.8 (19.7)	83.1 (20.9)		80.9 (21.1)	82.0 (21.9)
Body mass index, kg/m^2		29.8 (6.2)	29.7 (6.1)		29.3 (6.0)	30.3 (6.5)		29.6 (5.7)	30.0 (6.4)		29.3 (6.3)	29.7 (6.3)
Systolic blood pressure, mmHg		135.5 (16.6)	135.8 (16.8)		136.2 (17.2)	136 (17.4)		139.9 (18.7)	139.5 (18.7)		139.2 (17.7)	139.3 (16.4)
Diastolic blood pressure, mmHg		72.3 (10.3)	76.6 (10.7)		77.6 (10.8)	77.1 (9.6)		78.4 (10.9)	78.5 (10.5)		79.6 (9.9)	78.4 (9.6)
HbA1c, %		7.2 (1.8)	7.0 (1.6)		7.0 (1.6)	6.9 (1.6)		7.1 (1.7)	7.1 (1.8)		7.3 (1.8)	7.4 (1.9)
eGFR, ml/min per 1.73 m^2		44.9 (12.2)	44.4 (12.4)		43.6 (12.6)	43.9 (12.1)		41.5 (11.5)	43.0 (13.2)		41.5 (13.7)	41.1 (12.1)
Hemoglobin, g/L		129.5 (16.8)	130 (17.0)		129.7 (17.8)	129.9 (18.1)		129.1 (18.9)	127.4 (17.6)		123.1 (19.9)	123.3 (18.5)
Baseline ACE inhibitor/ARB use, n (%)		870 (98.8)	664 (97.4)		424 (97.7)	423 (98.8)		30 (98.7)	406 (98.1)		275 (98.9)	427 (97.5)
Baseline diuretic use, n (%)		401 (45.5)	296 (43.4)		179 (41.2)	195 (45.6)		171 (44.4)	196 (47.3)		135 (48.6)	200 (45.7)

Data are presented mean (SD), unless otherwise indicated. ACE, angiotensin-converting enzyme; ARB, angiotensin-receptor blocker; CKD, chronic kidney disease; IQR, interquartile range; n, number; SD, standard deviation; UACR, urinary albumin–creatinine ratio. * Among those participants with a history of diabetes.

Supplementary Figure 1. Risk of the primary and kidney end point by baseline and albuminuria level at month 4
(A and B) Risk of the primary and kidney end point by baseline and albuminuria level at month 4 for the primary (A) and kidney (B) end points, respectively. UACR, urinary albumin–creatinine ratio.

A

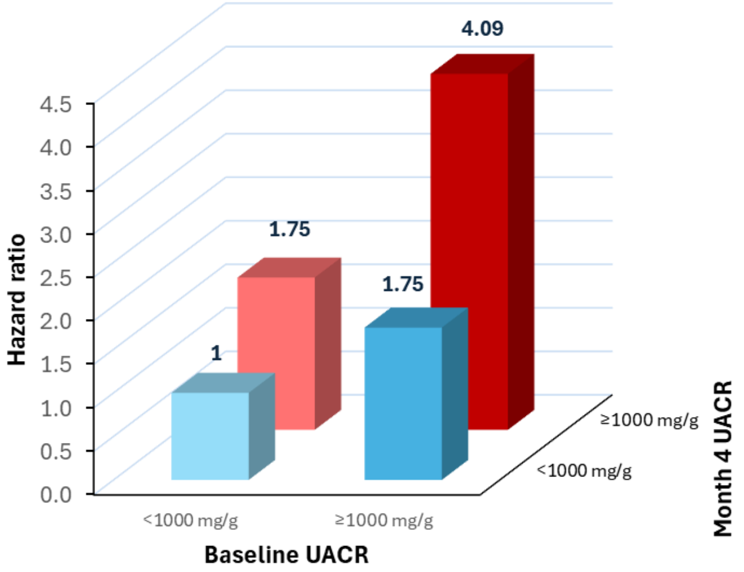

B

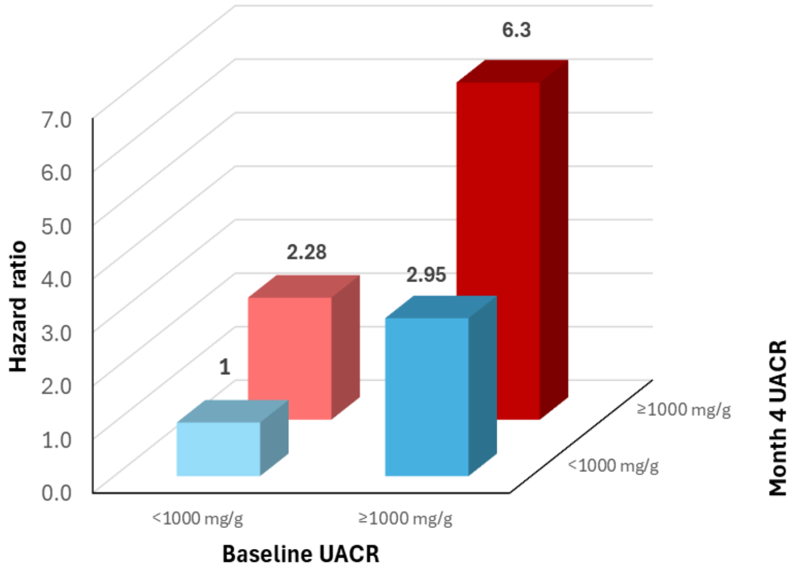

Supplementary Figure 2. Association of albuminuria at month 4 with primary and kidney end points in patients randomized to placebo or dapagliflozin, assessed by type 2 diabetes status
(A and B) Association of the residual albuminuria at month 4 with the primary end point for patients with and without diabetes, respectively, using one reference point (UACR category of 500 mg/g in the placebo group). (C and D) Association of the residual UACR at month 4 with the kidney end point for patients with and without diabetes, respectively, using one reference point (UACR category of 500 mg/g in the placebo group). Vertical lines represent 95% CI. CI, confidence interval; UACR, urinary albumin–creatinine ratio

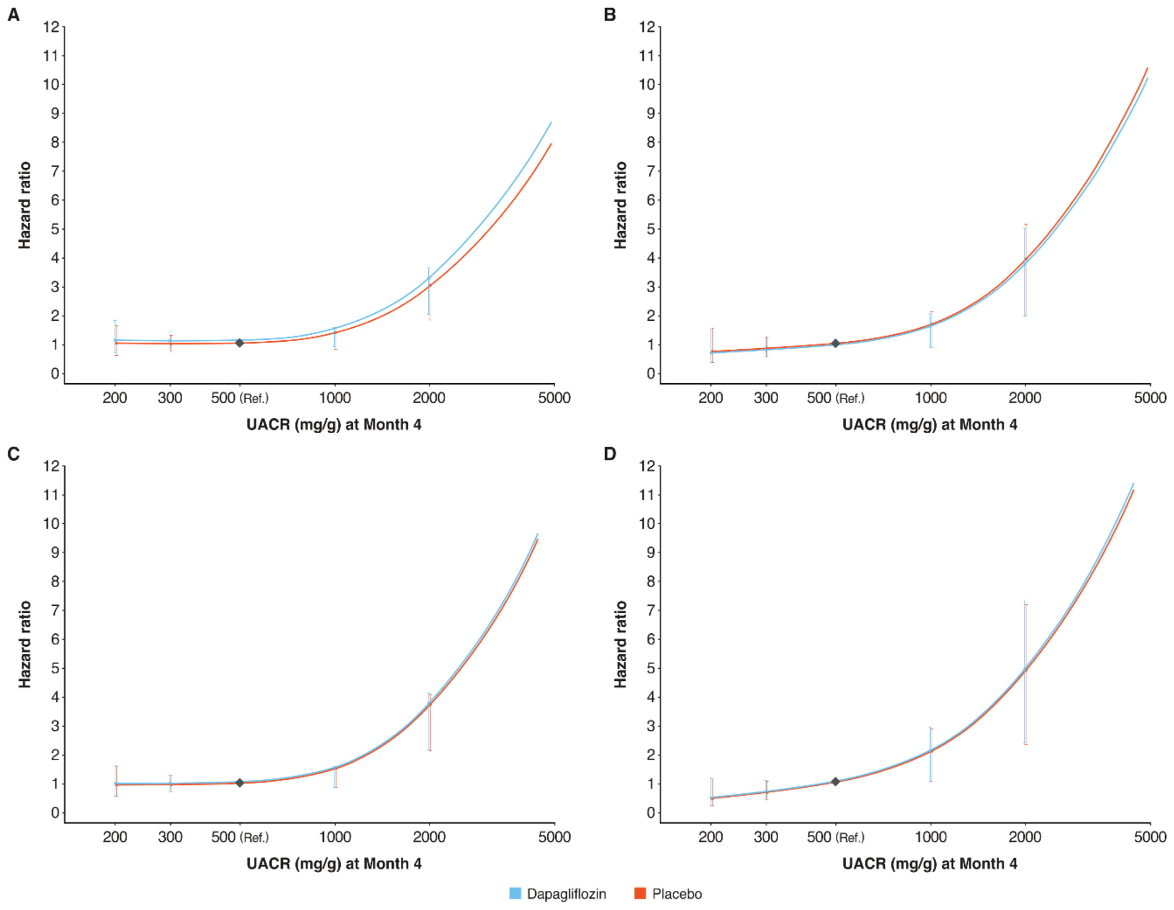

Supplement: Supplementary file 3 [file cjasn-19-1574-s003.pdf]
